# Extremely rare case of H-type gallbladder duplication coexistent with carcinoma: a case report and review of the literature

**DOI:** 10.1186/s40792-020-00953-3

**Published:** 2020-08-03

**Authors:** Takashi Furuhashi, Toshihiko Abe, Koichiro Sakata, Mayaka Noda, Tadafumi Fuchikami, Nobuhisa Ishikawa, Hiroyuki Fujito, Norihiro Onari, Tetsuo Hamada

**Affiliations:** 1 Department of Surgery, Japan Seafarers Relief Association Moji Ekisaikai Hospital, 1-3-1 Kiyotaki, Mojiku, Kitakyushu 801-8550 Japan; 2 Department of Gastroenterology, Japan Seafarers Relief Association Moji Ekisaikai Hospital, 1-3-1 Kiyotaki, Mojiku, Kitakyushu 801-8550 Japan; 3 Department of Radiology, Japan Seafarers Relief Association Moji Ekisaikai Hospital, 1-3-1 Kiyotaki, Mojiku, Kitakyushu 801-8550 Japan; 4 Department of Laboratory Medicine & Surgical pathology, Kyushu Railway Memorial Hospital, 2-1-1 Takada, Kitakyushu, 800-0031 Japan

**Keywords:** Gallbladder duplication, Adenocarcinoma, Intraoperative cholangiography, Near-infrared ray vision system, FDG-PET

## Abstract

**Introduction:**

Multiple gallbladders represent a rare congenital disorder, and coexistence with carcinoma is extremely rare, leading to a high possibility of misdiagnosis and surgical complications. In this study, a case was reported and the literature was reviewed.

**Case presentation:**

An 80-year-old woman was diagnosed with acute cholecystitis via ultrasonography and was successfully treated with antibiotics. After the patient’s biliary colic relapsed, she was referred to our hospital. Multiple imaging modalities revealed duplication of her gallbladder (H-type) and suggested coexistence with carcinoma. According to preoperative evaluations, we assumed the patient had stage IIIA disease, and cholecystectomy, cholangiography using a near-infrared ray vision system, and sectionectomy of segments 4a and 5 were performed. Contrary to the high standardized uptake values obtained by ^18^F-fluoro-2-deoxy-D-glucose positron emission tomography, gallbladder carcinoma was pathologically diagnosed as stage 0 mucosal cancer. Seven days after the operation, portal thrombosis of the posterior branch was revealed, and conservative therapy was indicated; satisfactory results were achieved. The patient was discharged 65 days after surgery. No recurrence was observed for 1 year after surgery.

**Conclusions:**

An extremely rare case of malignancy in a duplicated gallbladder was reported, and the literature was reviewed. Accurate estimations are feasible for diagnoses of multiple gallbladders, where correct evaluations are vital, especially in malignant cases. Because of the possibility of malignancy, resected accessory gallbladders should be scrutinized pathologically.

## Introduction

Multiple gallbladders (MGs) represent a rare congenital disorder, and coexistence with carcinoma is even more rare, resulting in a high risk of misdiagnosis and surgical complications. Although cases of MGs have been reported in 1 per 4000 autopsies [1], they are rarely recognized clinically. Boyden’s classification is the most widely accepted classification. He classifies double gallbladders into two types. Based on their relationship to the cystic duct, he describes “vesica fellea divisa” (a bilobed gallbladder that has one cystic duct) and “vesica fellea duplex” (true gallbladder duplication). True duplication is further subclassified into the “Y-shaped type” (two cystic ducts uniting before entering the common bile duct) and the “H-shaped or ductular type” (two cystic ducts enter individually into the common bile duct) [1]. Studies on MGs were identified in PubMed by searching for the words “multiple gallbladders,” “duplicated gallbladder,” “duplication of gallbladder,” “duplicate gallbladder,” and “double gallbladder” corresponding to the definition of true duplication according to Boyden’s classification. Furthermore, only five cases of gallbladder malignancy with MGs, except for MGs in a broad sense, have been reported in the English literature [2–6]; little is known about MGs associated with carcinoma. We herein report a rare case of MGs associated with carcinoma.

## Case presentation

An 80-year-old woman was admitted to her home doctor’s practice with abdominal pain and fever. She was diagnosed with acute cholecystitis via ultrasonography (US) and was successfully treated with antibiotics. When biliary colic relapsed, the patient was referred to our hospital. She had suffered from hypertension for 10 years. There were no special notes in her family history or from her relevant physical examination, nor were there any other significant clinical findings.

**Fig. 1:**
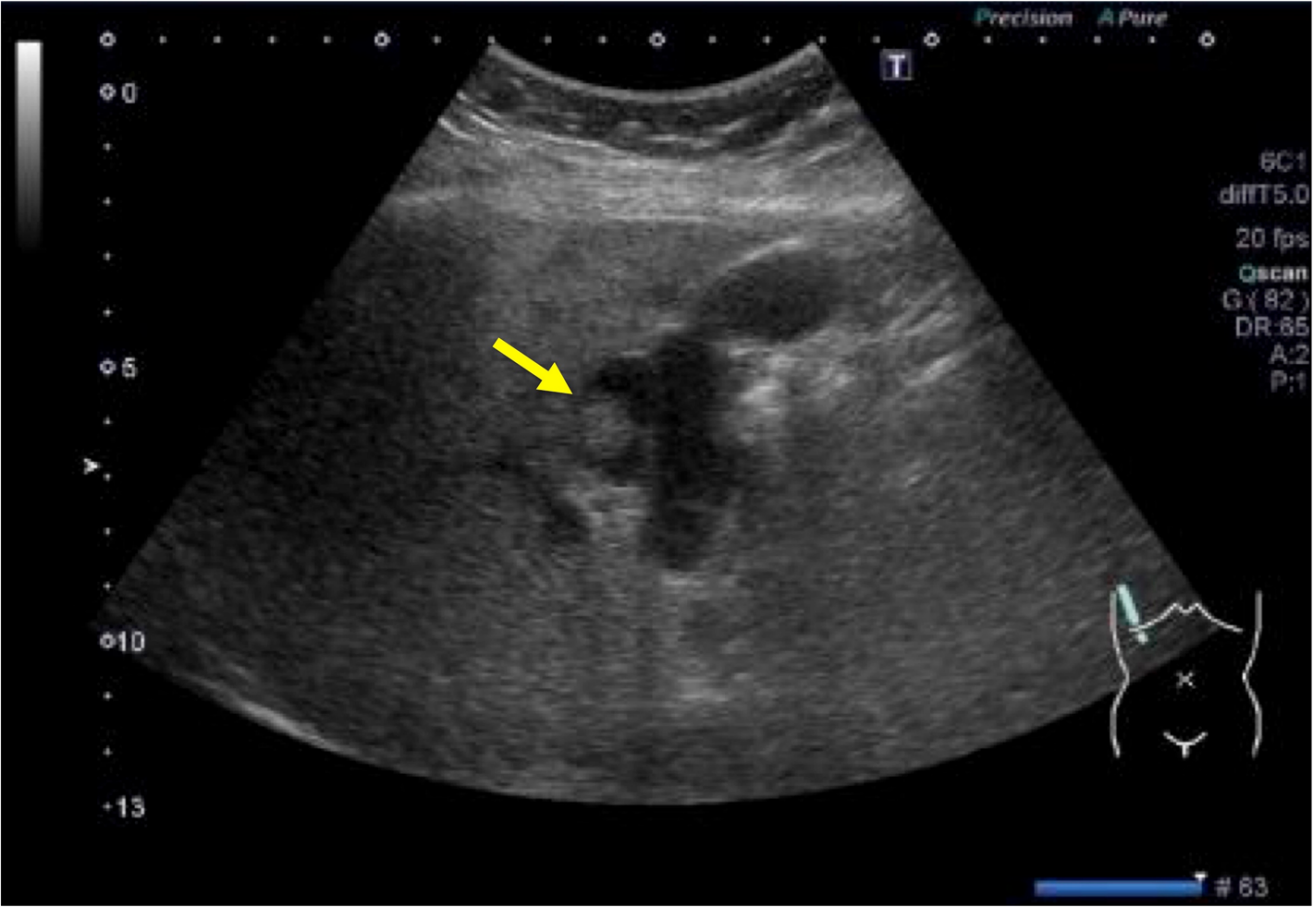
Ultrasonography imaging. The arrow shows carcinoma 1 cm in diameter in the proximal gallbladder with a base and obscure demarcation along the wall

**Fig. 2:**
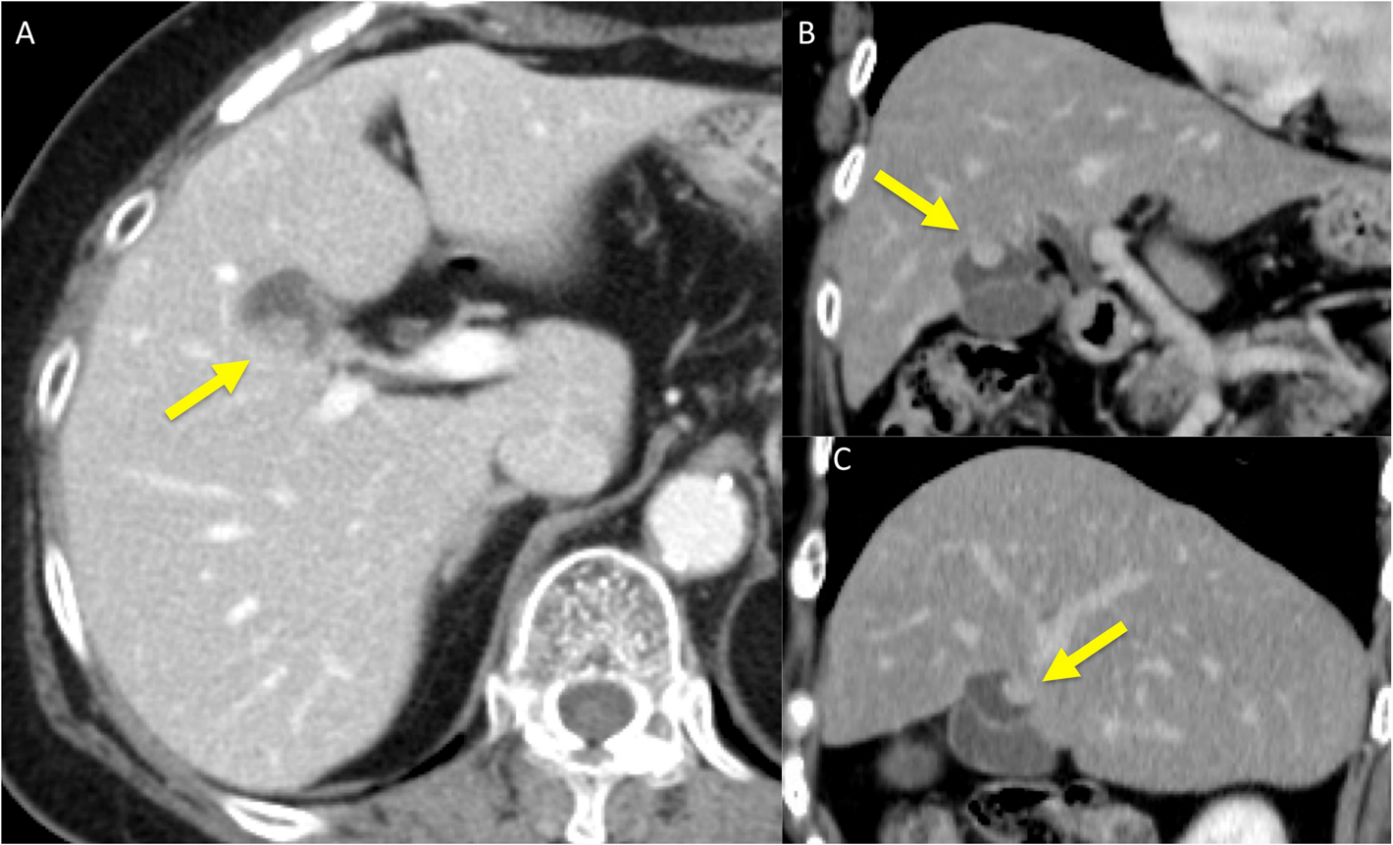
Abdominal enhanced CT imaging. Carcinoma in the proximal gallbladder, which was identified as the accessory gallbladder, shown by enhanced CT images. **a** Axial view. The low-density mass with a diameter of 1 cm was slightly enhanced (see the arrows). Direct liver invasion was less conspicuous on CT imaging. **b** Coronal view, and **c** sagittal view. Two separate cavities were clearly demonstrated

**Fig. 3:**
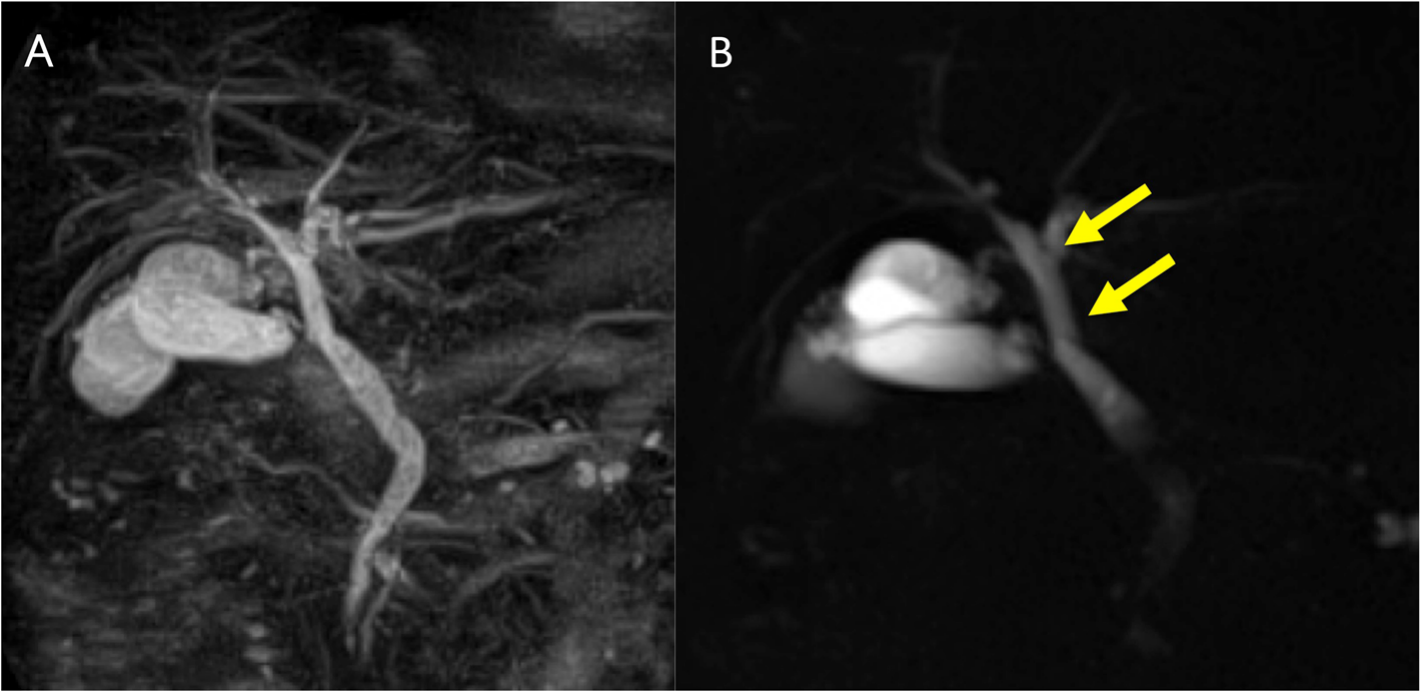
MRCP imaging. **a** 3D image and **b** 2D image. A definite H-type duplicated gallbladder was demonstrated. Both cystic ducts were identified as diverging individually from the common bile duct (see the arrows)

**Fig. 4:**
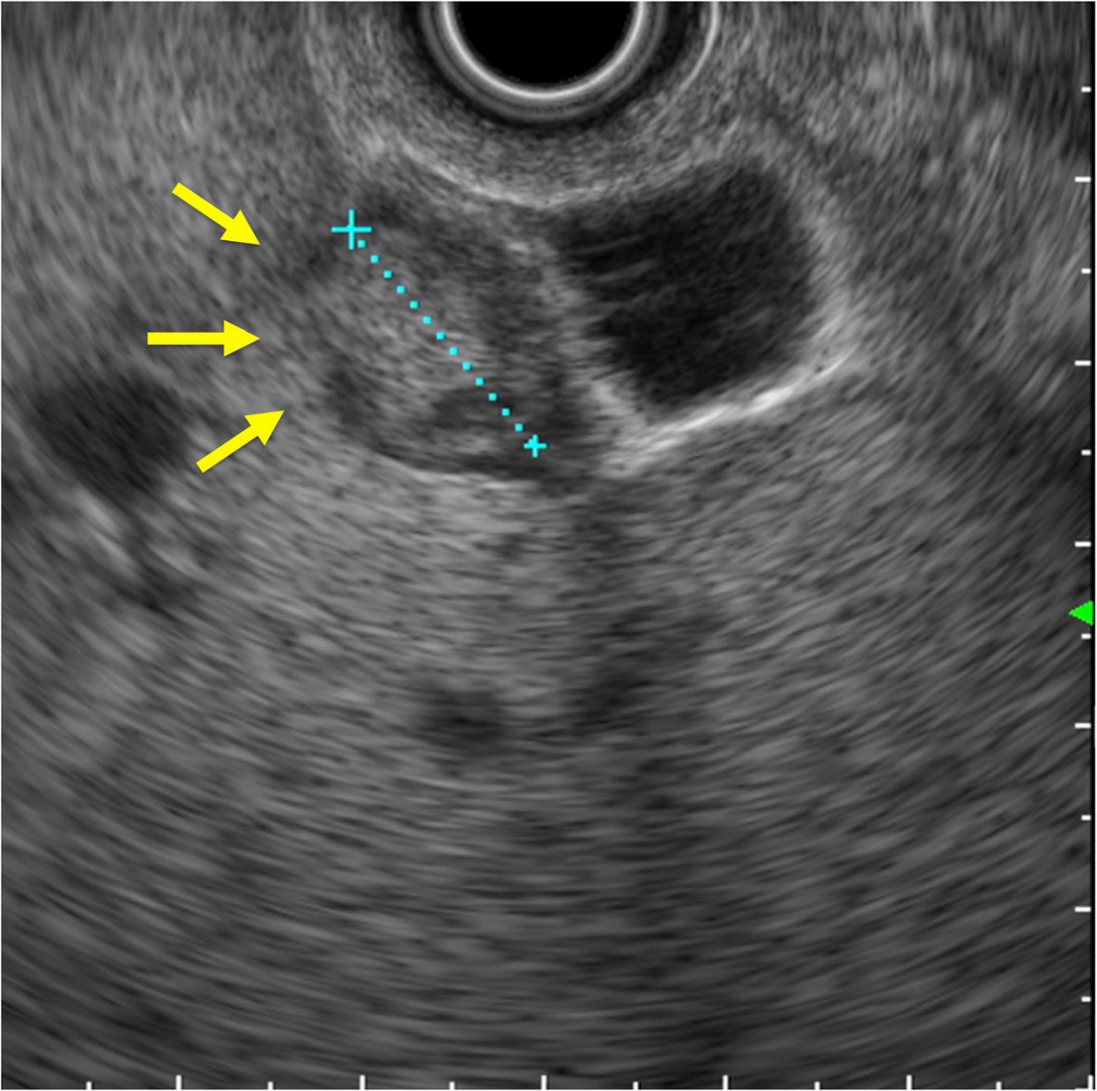
EUS imaging. Endoscopic ultrasonography demonstrated a solid tumor in the proximal gallbladder that seemed to invade the hepatic parenchyma adjacent to the gallbladder (see the arrows). The dotted line indicates the size of the mass

**Fig. 5:**
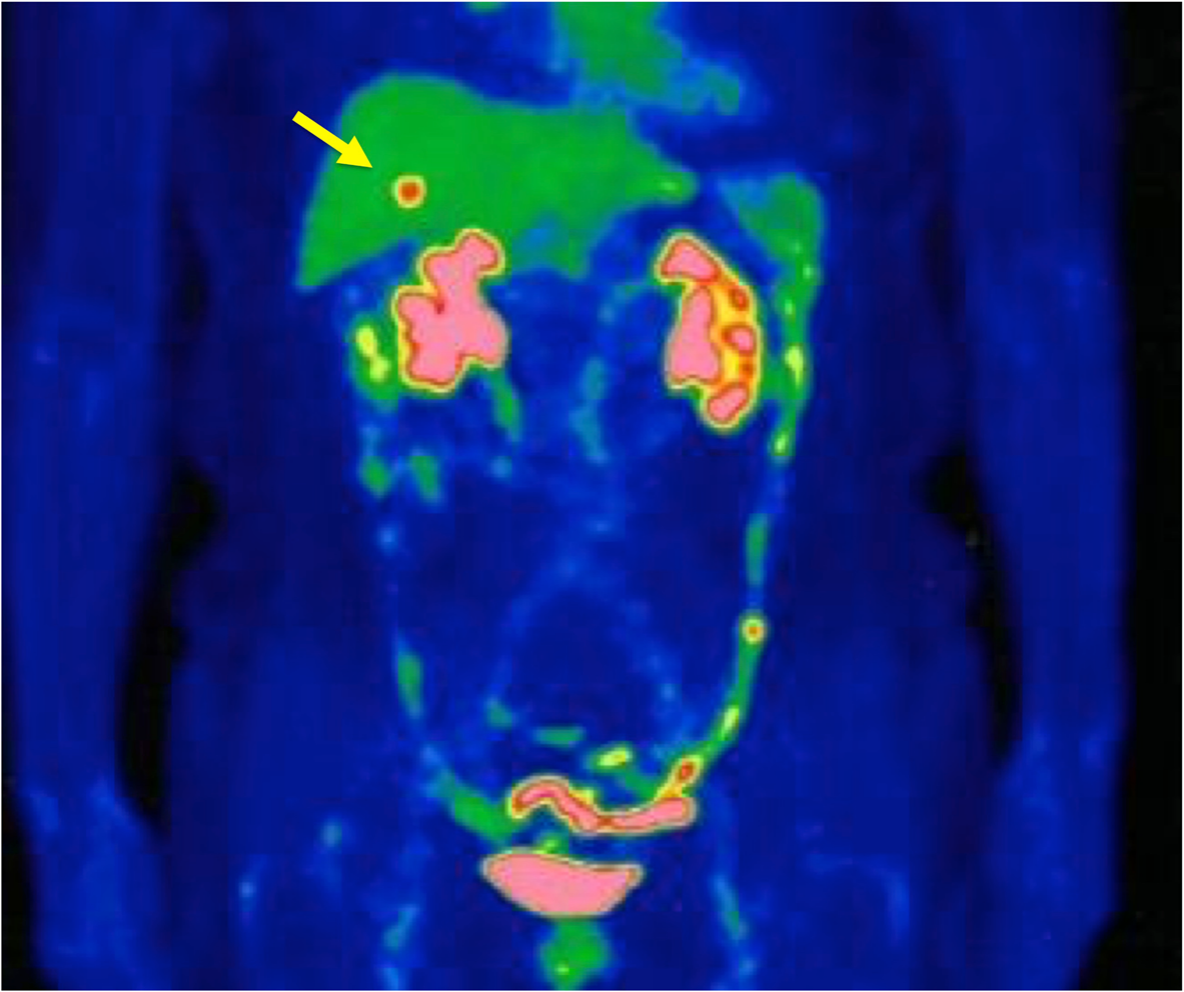
PET imaging. PET showed an abnormal FDG uptake (SUVmax 5.97) in the gallbladder (see the arrow). No other metastases were identified

**Fig. 6:**
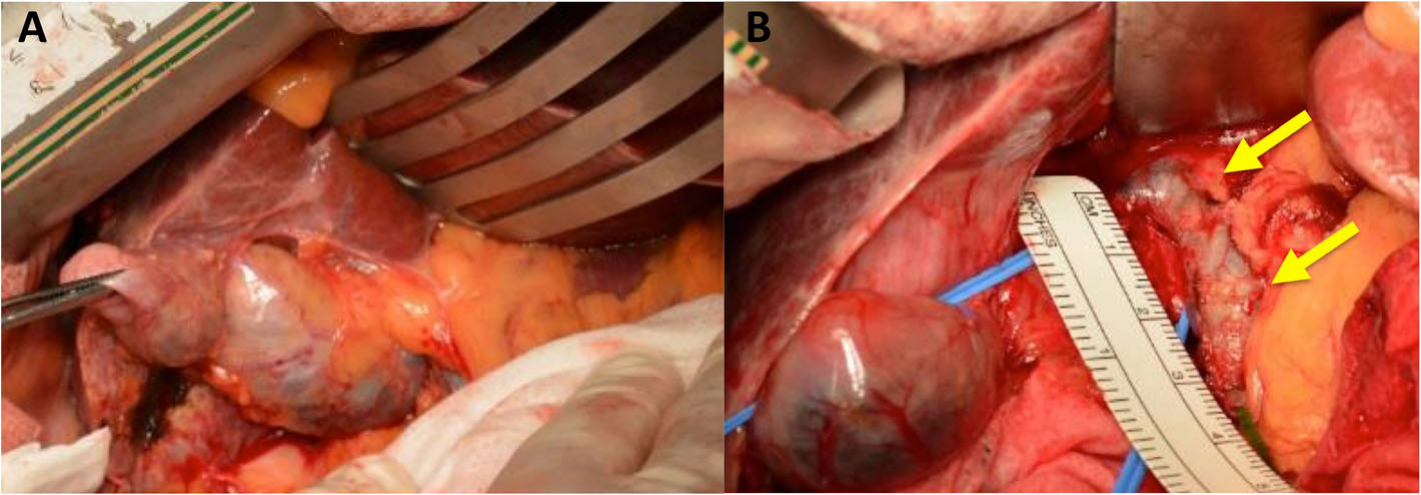
Intraoperative findings. **a** A double gallbladder was clearly identified. **b** A pair of cystic ducts (indicated by arrows) were confirmed

**Fig. 7:**
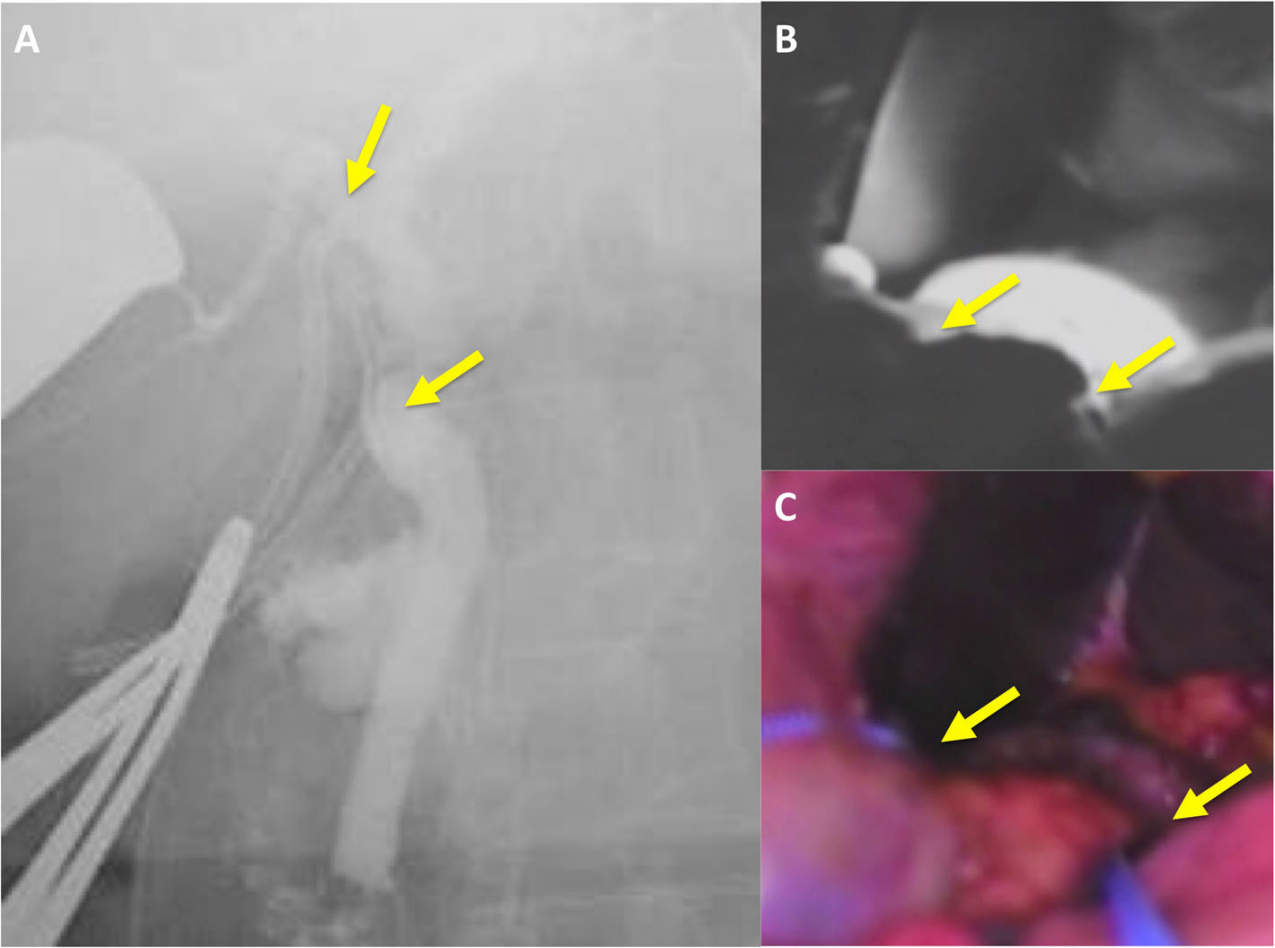
Intraoperative cholangiography. **a** Normal cholangiogram via iodine contrast agent. **b**, **c** Biliary images via a near-infrared ray vision system (Photo Dynamic Eye*®*: PDE). An intraoperative cholangiogram through the primary cystic duct showed the correct ductal anatomy. A pair of cystic ducts (indicated by arrows) were demonstrated

**Fig. 8:**
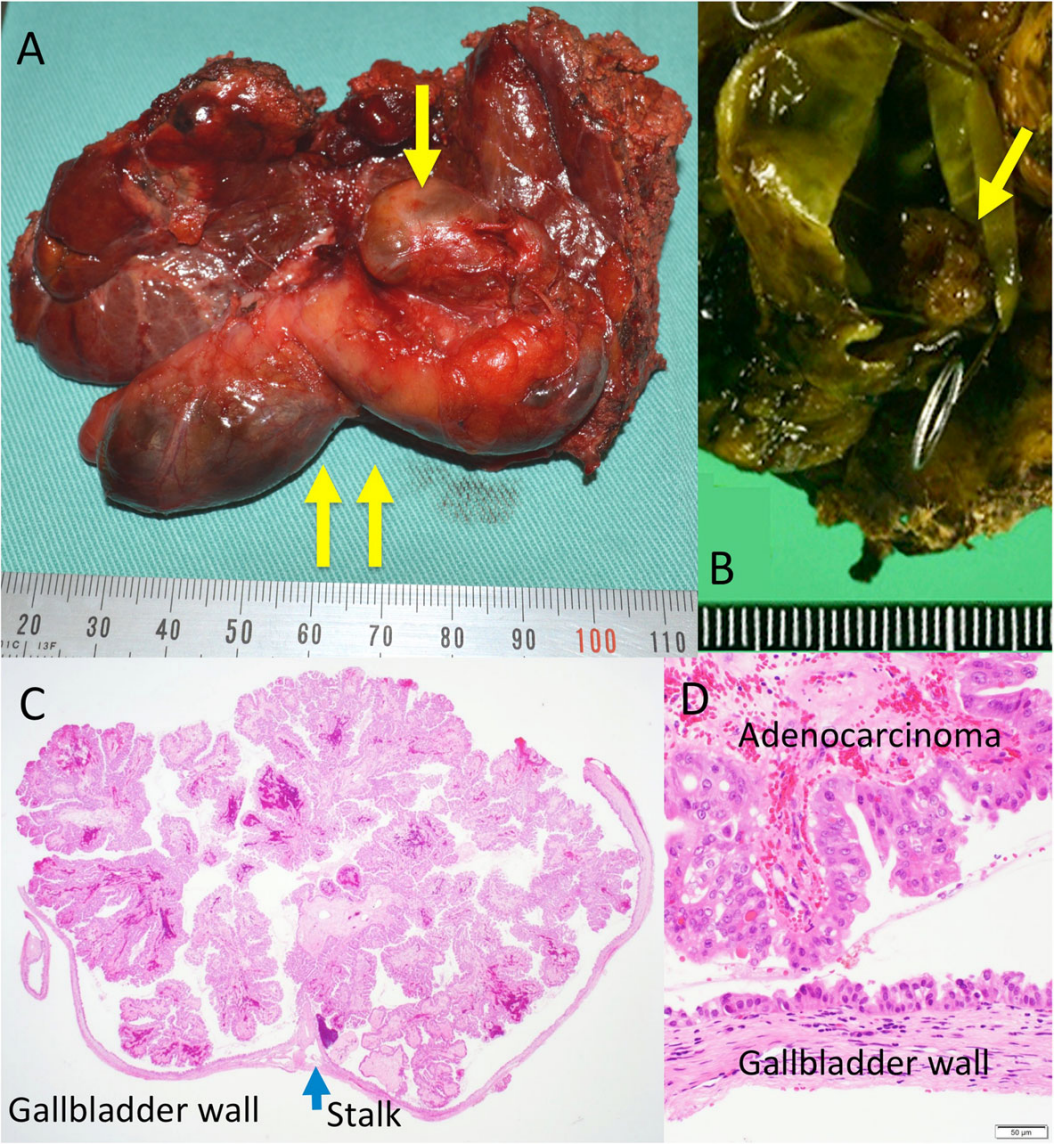
Macroscopic findings and histological findings of resected specimen. **a** Resected fresh specimen, proximal gallbladder identified as an accessory gallbladder containing carcinoma (single arrow), and distal gallbladder identified as a primary gallbladder (double arrows). **b** Formalin-fixed specimen, carcinoma in the proximal gallbladder identified as an accessory gallbladder (arrow). **c**, **d** Hematoxylin and eosin (H&E) staining at original magnification (× 20 and × 100). Pedunculated and non-invasive mucosal carcinoma had a fine stalk, and there was space between the mass and the gallbladder wall

First, images obtained by US showed gallbladder duplication and an elevated lesion with a diameter of 1 cm in the proximal portion, which was identified as an accessory gallbladder (Fig. 1). An abdominal computed tomography (CT) scan with enhancement revealed a low-density mass with early enhancement in the proximal gallbladder. Direct liver invasion was less conspicuous on CT imaging (Fig. 2). Magnetic resonance cholangiopancreatography (MRCP) revealed that both cystic ducts had diverged from the common bile duct individually, so an H-type duplicated gallbladder was considered according to Boyden’s classification (Fig. 3). Endoscopic ultrasonography (EUS) indicated a hypoechoic lesion with a ragged edge, and an increased central area signified penetration into the hepatic parenchyma through the gallbladder bed (Fig. 4). The maximum standardized uptake value (SUV) from ^18^F-fluoro-2-deoxy-D-glucose positron emission tomography (FDG-PET) was revealed as 5.97 at the accessory gallbladder. No other metastases were identified in any of the images (Fig. 5). The patient’s laboratory tests revealed a normal complete blood count and mild liver dysfunction with an indocyanine green plasma disappearance rate of 15% at 15 min. The serum carcinoembryonic antigen (CEA) level, carbohydrate antigen 19-9 (CA19-9) level, and serum SPan-1 antigen level were 2.2 ng/mL (normal range, < 5 ng/mL), 35.6 U/mL (normal range, < 37 U/mL), and 21 U/mL (normal range, < 30 U/mL), respectively. Consequently, the patient was diagnosed with advanced gallbladder carcinoma of the accessory gallbladder (H-type), stage IIIA (T3, N0, M0) according to the 8th edition of the UICC TNM classification of malignant tumors [7]. The total liver volume (TLV) was estimated to be 832 mL by three-dimensional volumetric simulation software (Synapse Vincent*®*; Fujifilm, Tokyo, Japan), and the volume for the limited resection of segments 4a and 5 was estimated to be 93 mL, which was equivalent to 11.2% of the TLV. A double gallbladder was clearly identified macroscopically after laparotomy (Fig. 6). Intraoperative cholangiography was performed in the primary cystic duct with a near-infrared ray vision system (Photo Dynamic Eye®: PDE, HAMAMATSU Photonics, Shizuoka, Japan), which revealed a pair of cystic ducts that had been diagnosed preoperatively (Fig. 7). Intraoperative frozen-section examination of the accessory cystic duct was submitted. Although radical dissection of the lymph nodes in the hepatoduodenal ligament, including the no. 13 lymph nodes on the posterior surface of the pancreatic head, was performed during the examination while preserving the biliary tract using a near-infrared ray vision system, no enlarged lymph nodes were found. As several studies have addressed, a near-infrared ray vision system can be useful for visualizing the extrahepatic biliary anatomy during hepatobiliary surgery. The pathological report from the frozen specimen proved negative for carcinoma at the surgical margin, so sectionectomy of S4a and 5 with MGs was performed. The operation time was 325 min, and the intraoperative blood loss was 550 mL. No blood transfusion was needed. Examination of the resected specimen showed that the gallbladder wall was thin, and the demarcation between the wall and the mass was clear. The mass was pedunculated with a fine stalk, and there was space between the mass and the gallbladder wall. Pathological examination of the surgical specimen revealed biliary type adenocarcinoma (WHO); papillary adenocarcinoma; papillary expanding type (9 × 7 mm); pTis (M); int, ly0; v0; ne0; pEM0; pCM0; pPV0; pA0; pR0; and pN0 (0/7). The disease progressed through stages Tis, N0, and M0 and ultimately to stage 0 (according to the 8th edition of the UICC TNM classification of malignant tumors [7]) (Fig. 8). Seven days after the operation, portal thrombosis of the posterior branch was revealed, and conservative therapy was indicated; satisfactory results were achieved. The patient was discharged 65 days after surgery. No recurrence was observed for 1 year after surgery.

## Discussion

Cases of MGs are reported in 1 per 4000 autopsies [1], and anatomical recognition of the type of MGs is essential. Multiple imaging modalities, such as US, CT, MRCP, and endoscopic retrograde cholangiopancreatography (ERCP), can correctly diagnose MGs. Darnis et al. reviewed data from 181 patients extracted from 153 studies published between 1990 and 2017 [8]. They found that MGs were diagnosed prior to surgery in 76% of patients. Ozaki et al. claimed that the definitive diagnosis of a duplicated gallbladder could only be made intraoperatively [9]. Anatomical recognition resulted in the correct surgical procedure without complications such as bile duct injury or leakage.

Raymond and Thrift reported the first case in 1956 [2], and only five cases of gallbladder malignancy associated with MGs, except for MGs in a broad sense, have been reported in the English literature. These five reports and our case are summarized in Table 1. Roeder et al. reported triplication of the gallbladder with papillary adenocarcinoma in 1971 [3]. They removed the two gallbladders identified intraoperatively, and the third gallbladder was detected on the fourteenth postoperative day via T tube cholangiography. Kin et al. reported a case of gallbladder duplication with advanced adenocarcinoma in 1996 [4]. They noted that magnetic resonance imaging (MRI) was the most useful modality in revealing the cystic formation of the accessory gallbladder with packed stones in addition to the main gallbladder. In addition, celiac angiography demonstrated the unusual form of the intrahepatic artery that enveloped the cystic tumor, and they noted that it might be a diagnostic characteristic of accessory gallbladders. Kawanishi et al. reported carcinoma visualized with abnormal accumulation by FDG-PET [5]. Well-differentiated tubular adenocarcinoma with infiltration into the submucosal layer was observed in the resected accessory gallbladder. Recently, Chen et al. reported a case of adenosquamous carcinoma in 2018 [6]. In their case, cholangiocarcinoma coexisted at the end of the common bile duct. Safioleas et al. stated that the incidence of gallstones in MG was similar to that in single gallbladders [10]. Additionally, Darnis et al. and Hurst et al. stated that there was no evidence of an increased risk for malignancy in MGs [8, 11]. However, as Kin et al. pointed out [4], attention must be paid to the fact that carcinoma in those 5 cases and in our case arose only from the accessory gallbladder. Although a higher frequency of malignancy in MGs that in single gallbladders has not been confirmed, resected accessory gallbladders should be scrutinized pathologically.

**Table 1:** Summary review of case report articles on multiple gallbladders with carcinoma

Author	Year	Sex	Age (year old)	Type of duplication	Preoperative imaging modalities	Location of carcinoma	Preoperative diagnosis of MG/carcinoma	Histological description	Stone	Procedure
Raymond et al.	1956	F	53	H-type, ductular type	None	Accessory gallbladder	No/no	Papillary adenocarcinoma	(−)	Cholecystectomy
Roeder et al.	1971	M	36	H-type (triplication), right trabecular type	None	Accessory gallbladder (second gallbladder)	No/no	Papillary adenocarcinoma	(−)	Cholecystectomy
Kin et al.	1996	F	50	H-type, left trabecular type	US, CT, MRI, cholangiography via PTBD tube, angiography	Accessory gallbladder	No/yes	Poorly differentiated adenocarcinoma (malignant cells had spread to the surrounding tissues both directly and via lymph vessels in the Grisson’s sheath)	(−)	Extended right hepatectomy
Kawanishi et al.	2010	M	75	H-type, left trabecular type	US, CT, MRCP, ERCP, PET, cholangiography via ENBD tube	Accessory gallbladder	Yes/yes	Well differentiated tubular adenocarcinoma (with infiltration into the submucosal layer)	(+)	Cholecystectomy
Chen et al.	2018	F	58	H-type, ductular type	US, CT, MRCP	Accessory gallbladder	Yes/yes	Adenosquamous carcinoma (2.5 × 2.0 × 1.5 cm), coexistence of cholangiocarcinoma, at the end of the common bile duct (1.2 cm)	(−)	Not described
Our case	2020	F	80	H-type, ductular type	US, CT, MRCP, EUS, PET	Accessory gallbladder	Yes/yes	Papillary adenocarcinoma (mucosal carcinoma, 0.9 × 0.7 cm)	(−)	Segmentectomy of S4a and 5 with radical dissection of the lymph nodes in the hepatoduodenal ligament

*M* male, *F* female, *PTBD* percutaneous transhepatic biliary drainage, *ENBD* endoscopic nasobiliary drainage

Recently, diagnostic precision has been improved by EUS, which has been suggested to be accurate for the clinical staging of gallbladder carcinoma [12–14]. However, we incorrectly interpreted our EUS findings to be reflective of advanced carcinoma.

The SUV max evaluated by FDG-PET was 5.97 in our case, which was supposed to be indicative of malignancy; the patient’s past history of cholecystitis was taken into account because an experimental animal model indicated that the SUV max would be normalized within 1 week of the onset of acute inflammation [15]. However, in our case, the serum C-reactive protein level was almost in the normal range on the day of the FDG-PET examination. In the case of Kawanishi et al, the SUV max evaluated by FDG-PET was 4.8, despite the presence of submucosal carcinoma [5]. Although we recognized the importance of FDG-PET, we could not reconcile the differences in these results. It is reasonable to speculate that remnants of inflammation or tumor volume but not a high level of malignancy affect the SUV max as evaluated by FDG-PET.

In the extremely rare case of malignancy, correct cancer staging is critical. This case presented as stage 0 pathologically but was overdiagnosed as clinical stage IIIA based on an incorrect interpretation of the EUS findings and an equivocal SUV max that was probably affected by previous inflammation. In cases of MGs with malignancy, attention should be paid to accurate staging in cholecystitis patients. However, few reports are available on this issue, so further studies are needed to assess the coexistence of gallbladder duplication and carcinoma.

## Conclusions

We reported an extremely rare case of malignancy in a duplicated gallbladder. Accurate estimation is feasible for the diagnosis of MGs. In cases of malignancy, especially in cases with inflammation, correct cancer staging is vital. Because of the possibility of malignancy, resected accessory gallbladders should be scrutinized pathologically.

## Data Availability

All data generated or analyzed during this study are included in this article.

## Abbreviations

MGs: Multiple gallbladders
US: Ultrasonography
CT: Computed tomography scan
MRCP: Magnetic resonance cholangiopancreatography
EUS: Endoscopic ultrasonography
SUV: Standardized uptake value
FDG-PET: ^18^F-fluoro-2-deoxy-D-glucose positron emission tomography
CEA: Carcinoembryonic antigen
CA19-9: Carbohydrate antigen 19-9
TLV: Total liver volume
WHO: World Health Organization
ERCP: Endoscopic retrograde cholangiopancreatography
MRI: Magnetic resonance imaging
PTBD: Percutaneous transhepatic biliary drainage
ENBD: Endoscopic nasobiliary drainage
